# Automated Prediction of Photographic Wound Assessment Tool in Chronic Wound Images

**DOI:** 10.1007/s10916-023-02029-9

**Published:** 2024-01-16

**Authors:** Nico Curti, Yuri Merli, Corrado Zengarini, Michela Starace, Luca Rapparini, Emanuela Marcelli, Gianluca Carlini, Daniele Buschi, Gastone C. Castellani, Bianca Maria Piraccini, Tommaso Bianchi, Enrico Giampieri

**Affiliations:** 1 Department of Physics and Astronomy, University of Bologna, 40127 Bologna, Italy; 2 Data Science and Bioinformatics Laboratory, IRCCS Institute of Neurological Sciences of Bologna, 40139 Bologna, Italy; 3 Dermatology Unit, IRCCS Azienda Ospedaliero-Universitaria di Bologna, 40138 Bologna, Italy; 4 Department of Medical and Surgical Sciences, University of Bologna, 40138 Bologna, Italy; 5 eDIMESLab, Department of Medical and Surgical Sciences, University of Bologna, 40138 Bologna, Italy; 6 Native Medica s.r.l., Native Medica, 40138 Bologna, Italy

**Keywords:** PWAT, Image analysis, Wound healing, Computer vision, Clinical decision support system

## Abstract

Many automated approaches have been proposed in literature to quantify clinically relevant wound features based on image processing analysis, aiming at removing human subjectivity and accelerate clinical practice. In this work we present a fully automated image processing pipeline leveraging deep learning and a large wound segmentation dataset to perform wound detection and following prediction of the Photographic Wound Assessment Tool (PWAT), automatizing the clinical judgement of the adequate wound healing. Starting from images acquired by smartphone cameras, a series of textural and morphological features are extracted from the wound areas, aiming to mimic the typical clinical considerations for wound assessment. The resulting extracted features can be easily interpreted by the clinician and allow a quantitative estimation of the PWAT scores. The features extracted from the region-of-interests detected by our pre-trained neural network model correctly predict the PWAT scale values with a Spearman's correlation coefficient of 0.85 on a set of unseen images. The obtained results agree with the current state-of-the-art and provide a benchmark for future artificial intelligence applications in this research field.

## Introduction

Due to the average population age increase, more dermatologist specialists are involved in wound management [1]. Wound healing is a complex process, and optimal wound assessment is essential for their management; choosing the most appropriate therapeutic approach can reduce healing times, and thus alleviate the healthcare system's economic burden [2]. An incorrect wound assessment model can lead to prolonged wound healing [3] and decrease patient compliance. The correct classification of acute and chronic ulcers is essential both at diagnosis and in follow-up. A growing number of centers resort to archiving clinical images with methodical and instrumental-assisted continuous monitoring to ascertain whether the healing process is proceeding correctly or not and then to determine prognosis and correct treatment [4].

The entire clinical evaluation process relies on the experience and subjectivity of the clinicians, introducing a not negligible inter- and intra- operator variability [5]. The introduction of wound assessment tools aims to reduce these effects, providing a series of standardized criteria for the quantitative description of the wound status and response to the treatments. One of the most popular in the dermatological practice is the Bates-Jensen Wound Assessment Tool (BWAT) [6], which consists of 13 items that assess wound size, depth, edges, undermining, necrotic tissue type, amount of necrotic, granulation and epithelialization tissue, exudate type and amount, surrounding skin color, edema, and induration. The items are represented as Likert scales with values ranging from 1 to 5, associated with the unhealthiest attribute of each of them. The use of the BWAT requires the evaluation of the wound online, i.e., during clinical practice, since many of the items can be quantified only by manual operations on the lesion area. For this reason, automated solutions for the quantification of this score are not applicable and it is impossible its posterior editing or adjustment. To address these issues, the Photographic Wound Assessment Tool (PWAT) was introduced in 2000 [7]. The PWAT score, indeed, aims to quantify the wound status starting from photos acquired during the clinical practice and involving item-scores inferable directly by the picture. The PWAT includes only a subset of the full list of items described by the BWAT, but it has already proved its effectiveness and robustness for clinical applications [6, 7].

Despite the introduction of standardized assessment tools, the intrinsic subjectivity of the clinicians in the grading process continues to play a key role. The Likert format of the scale items, indeed, poses some constraints in the evaluation, but it forces the quantification of wound features which can be determined only by human intervention. The possibility to obtain a completely objective estimation of wound status can be addressed only by introducing an agnostic mechanical component guided by the ever-growing artificial intelligence solutions. The application of artificial intelligence models to medical image analysis already showed remarkable results [8–11], proving its effectiveness in guiding and facilitating clinical practice [12, 13]. According to the forementioned wound assessment tools, automated solutions for their estimations have been already proposed in literature [8, 14, 15], providing hints about their mathematical formalization but without a detailed analysis of the related features. The current trend of the medical image analysis, is based on the use of deep learning models for the prediction of the clinical outcomes, making harder the understanding of the relevant clinical features. Also in the context of the PWAT prediction, several approaches have already been proposed in literature, but only based on neural network models [14, 16, 17]. In our previous work [18]*,* we trained a deep learning model to perform semantic segmentation of wound region-of-interests (ROIs) from digital images. Here, we extend the model to automatically predict the PWAT scores from the identified wound areas.

The PWAT includes items belonging to both the wound and peri-wound areas, so we adapted our model predictions to obtain both these ROIs, we then proposed a novel set of textural and morphological features mimicking the clinician’s manual evaluation. According to these principia, all the proposed features are strictly connected to the wound appearance and completely human interpretable, guaranteeing their possible application during the clinical practice. We finally use this set of features to feed a penalized regression model for the prediction of the PWAT scale value, testing the effectiveness and robustness of our model on an independent subset of images. To the best knowledge of the authors, our work represents the first attempt to automatically predict the PWAT score on smartphone images, using a combination of standard and radiomic image features.

## Materials and methods

### Patient selection

In this work we analyzed the images belonging to the *Deepskin* dataset [18]. The images were acquired using smartphone cameras during routine dermatological examinations by the Dermatology Unit at IRCCS Sant'Orsola-Malpighi University Hospital of Bologna. The images were retrieved from charts of subjects who gave their voluntary consent to research. The Local Ethics Committee approved the study and carried it out in accordance with the Declaration of Helsinki. The data acquisition protocol was approved by the Local Ethics Committee (protocol n° 4342/2020 approved on 10/12/2020) according to the Helsinki Declaration.

We collected 474 patients over two years (from March 2019 to September 2021) at the center with 1564 wound images. A smartphone digital camera (Dual Sony IMX 286 12MP sensors with 1.25 µm pixel size, 27 mm equivalent focal length, F2.2 aperture, Laser-assisted AF, DNG Raw capture) acquired the raw images under uncontrolled illumination conditions, various backgrounds, and image expositions for clinical usage. The involved patients belonged to a heterogeneous population, including samples with ulcers at different healing stages and anatomical positions.

In this work, we used a subset of data extracted from the *Deepskin* dataset, composed of 612 images. This subset includes 324 males (52.9%) and 288 females (47.1%), with an average age of 77 ± 17 and 71 ± 17, respectively. Therefore, the involved population was balanced according to sex and biased towards higher age, as expected in any dermatological wound dataset.

The heterogeneity of the population in terms of wound severity was preserved also in the considered subset. The corresponding PWAT distribution, indeed, ranges from a minimum of 2 to a maximum of 24 with an average of 15 ± 3. Also in this case, the bias related to relatively higher value of PWAT is considered acceptable in relation to the clinical problem and intrinsically due to the necessary presence of wound in each image.

### Clinical scoring of images

Two trained clinicians evaluated the 612 images independently. The clinicians scored each image according to the PWAT grading scale. For a robust estimation of the PWAT score, the quantification of the related sub-items was performed during the image acquisition (online evaluation), i.e., monitoring the actual state of the wound. We chose the PWAT scale since it is a standard reference for wound assessment in clinical practice, and its automation can easily encourage the clinicians' community to use our method.

All the clinicians scored the wounds in the same physical space, with the same source of illumination and without time limits. Each wound evaluation was reviewed according to the photo acquired during the clinical practice (offline evaluation), discarding all doubtful cases. During the offline evaluation, the images were displayed using a computer monitor (HP Z27 UHD 4 K, 27") with 3840 × 2160 resolution. The same screen color and brightness were used for the clinicians' evaluation.

### Image processing pipeline

**Fig. 1:**
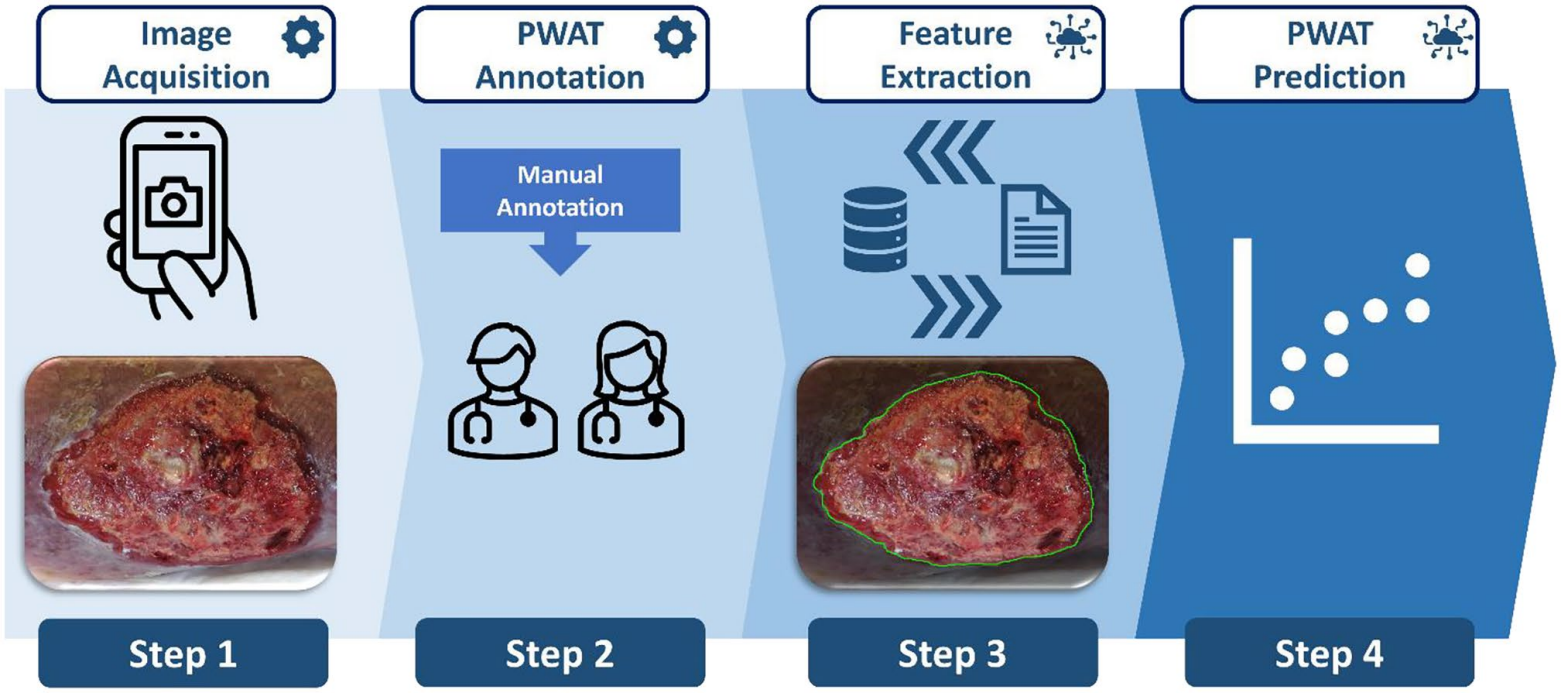
Schematic representation of the pipeline. (Step 1) The image is acquired by the smartphone (*Deepskin* dataset) during clinical practice. (Step 2) Two expert clinicians performed the manual annotation of the PWAT score associated to each wound, considering the status of the lesion and peri-lesion areas. (Step 3) The neural network model trained on the *Deepskin* dataset performs the automated segmentation of the wound area. Focusing on the wound and peri-wound areas (obtained by image processing analyses), a set of features for the quantification of textures and morphology of the lesion are extracted. (Step 4) A regression model based on the features extracted from the images is tuned for the automated prediction of the PWAT scores. While Step 1 and Step 2 requires the human intervention, by definition, the second half of the pipeline automatically performed the analysis. We would like to stress that the first two steps are mandatory for the training of the automated solution but are discarded during real clinical applications

The proposed image processing pipeline is composed of a series of independent and fully automated steps (ref. Fig. 1):

- **Step 1. **Image acquisition using smartphone camera during clinical practice.
- **Step 2. **Manual annotation of the wound status according to the PWAT scores by the two expert clinicians, providing the ground truth required for the training of the automated model.
- **Step 3. **Automated identification of the wound and peri-wound areas using the neural network model trained on the *Deepskin* dataset; extraction of human interpretable features for the quantification of the PWAT items and wound status.
- **Step 4.** Prediction of the PWAT score via weighted combination of the identified features.

The first step of processing involves segmenting the wound area from the background. For the automated segmentation of the images, we used our previously published convolutional neural network model: the details about the model implementation and its performances on the *Deepskin* dataset are discussed in our previous work [18]. The efficiency of wound segmentation is crucial for identifying the regions of interest on which perform the subsequent feature extraction. The segmentation masks generated by our neural network model involve only the wound bed areas, while several PWAT sub-items concern scores describing the peri-wound boundaries. To overcome this issue, we extended each wound mask using a combination of morphological operators, extracting a second mask related to the only peri-wound areas (ref. Wound segmentation section).

In the second step, we performed a features extraction from the areas identified by the segmentation model and the peri-wound masks. We extracted a set of standard image features based on different color spaces (RBG and HSV), redness measurements based on quantities already proposed in literature [19, 20], and the Haralick's textural features [21] for the quantitative description of wound morphology (ref. Wound features section for details about them).

In step three, the extracted set of features was used to feed a penalized regression model for the prediction of the final PWAT scale value.

## Calculation

### Wound segmentation

The definition of the wound area gives the principal limit of the *Deepskin* dataset. Since there is not a standardized set of criteria for the wound area definition, its reliability is left to the clinical needs. In our previous work, we trained a convolutional neural network model to segment the regions involving only the wound bed areas. In contrast, the Peri-ulcer Skin Viability and Edges items for the PWAT estimation involve the description of the peri-wound area, which is excluded by our segmentation mask.

**Fig. 2:**
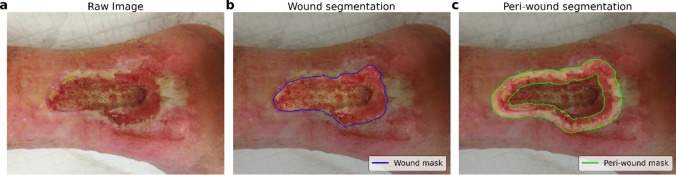
Example of segmentation masks used for wound identification. **a** Raw image extracted from *Deepskin* dataset. **b** Wound segmentation mask generated by automated neural network model. **c** Peri-wound segmentation mask obtained applying morphological operators on wound mask

In this work, we implemented a second step of automated image processing for the identification of the peri-wound areas, starting from the segmentation masks generated by our model. Using a combination of erosion and morphological dilation operators, keeping fixed the size of the structuring element (kernel) involved, we extracted for each image the associated peri-wound mask, i.e.

$${M}_{peri-lesion}=\left({M}_{lesion}\oplus k\right)-\left({M}_{lesion}\ominus k\right)$$

where ⊕ and ⊖ denote the dilation and erosion operators between the wound mask (M) and the kernel k, respectively. We used an ellipse shape for the kernel, with a dimension of 3 × 3. An example of the resulting image processing is shown in Fig. 2.

### Wound features

The quantification of the items related to the PWAT estimation involves both the wound and peri-wound areas. Since only 1/8 of PWAT sub-items involves the peri-wound area, we independently performed the features extraction on both the ROIs. In this way, we aimed to maximize the informative power of the features extracted from the wound area, minimizing the putative confounders, but preserving the information related to the peri-wound area.

#### Color features

We extracted the average and standard deviation of *RGB* channels for each wound and peri-wound segmentation. This set of measures aims to quantify the appearance of the wound area in terms of redness and color heterogeneity.

We converted each masked image into the corresponding *HSV* color space. For each channel, we extracted the average and standard deviation values. The *HSV* color space is more informative than the *RGB* one since it takes care of different light exposition (saturation). In this way, we monitored the various conditions in which the images were acquired.

Both these two sets of features aim to quantify the necrotic tissue components of the wounds. The necrotic tissue, indeed, could be modeled as a darker component in the wound/peri-wound area, which alters the average color of the lesion. The *Necrotic Tissue type* and the *Total Amount of Necrotic Tissue* involve 2/8 items in the PWAT estimation.

#### Redness features

The primary information on the healing stage of a wound can be obtained by monitoring its redness (erythema) compared to the surrounding area. Several redness measurements are proposed in literature [22], belonging to different medical fields and applications. In this work, we extracted two measures of redness, validated in our previous work [23] on a different image processing topic.

The first measure was proposed by Park et al. [20], and involves a combination of the *RGB* channels, i.e.,

$$Rednes{s}_{RGB}=\frac{1}{n}\sum_{i=1}^{n}\frac{2{R}_{i}-{G}_{i}-{B}_{i}}{2\times \left({R}_{i}+{G}_{i}+{B}_{i}\right)}$$

where *R*, *G*, and *B* are the red, green, and blue channels of the masked image, respectively, the *n* value represents the number of pixels in the considered mask. This measure emphasizes the *R* intensity using a weighted combination of the three *RGB* channels.

The second measure was proposed by Amparo et al. [19], and involves a combination of the *HSV* channels, i.e.,

$$Rednes{s}_{HSV}=\frac{1}{n}\sum_{i=1}^{n}{H}_{i}\times {S}_{i}$$

where *H* and *S* represent the hue and saturation intensities of the masked image, respectively. This measure tends to be more robust against different image light expositions.

Both these features were extracted on the wound and peri-wound areas independently. Redness estimations could help to quantify the *Peri-ulcer Skin Viability*, *Granulation Tissue Type*, and *Necrotic Tissue Type*, which represent 3/8 items involved in the PWAT estimation.

#### Morphological features

We measured the morphological and textural characteristics of the wound and peri-wound areas by computing the 13 Haralick's features [21]. Haralick's features are becoming standard texture descriptors in multiple medical image analyses, especially in the Radiomic research field [24–28]. This set of features was evaluated on the grey-level co-occurrence matrix (GLCM) associated with the grayscale versions of the original images, starting from the areas identified by our segmentation models. We computed the 13 standard Haralick's features, given by energy, inertia, entropy, inverse difference moment, cluster shade, and cluster prominence. Using textural elements, we aimed to quantify information related to the *Granulation Tissue types* and *Amount of Granulation Tissue*, which are 2/8 items of the total PWAT score.

### Regression pipeline

We started the regression analysis by standardizing the distribution of the extracted features. Each distribution of features belongs to a different domain of values, and to combine them, we need to rescale all the values into a common range. We rescaled the distributions of features using their median values, normalizing according to the 1st and 3rd quantiles, i.e., a robust scaling algorithm, minimizing the dependency from possible outliers. Both medians and quantiles were estimated on the training set and then applied to the test set to avoid cross contamination.

Starting from the processed features, we used a penalized Lasso regression model [29] to predict the PWAT clinical scores. Lasso regression is a regularized linear regression variant with an additional penalization component in the cost function [30]. In our simulations, we used a penalization coefficient equal to 10^-2^. We split the complete set of data into train/test sets using a shuffled tenfold stratified cross-validation: in this way, we can ensure a balance between classes at each subdivision. The model was trained on a subset (90%) of data, and its predictions were evaluated on the remaining test set (10%), at each fold.

## Results

**Fig. 3:**
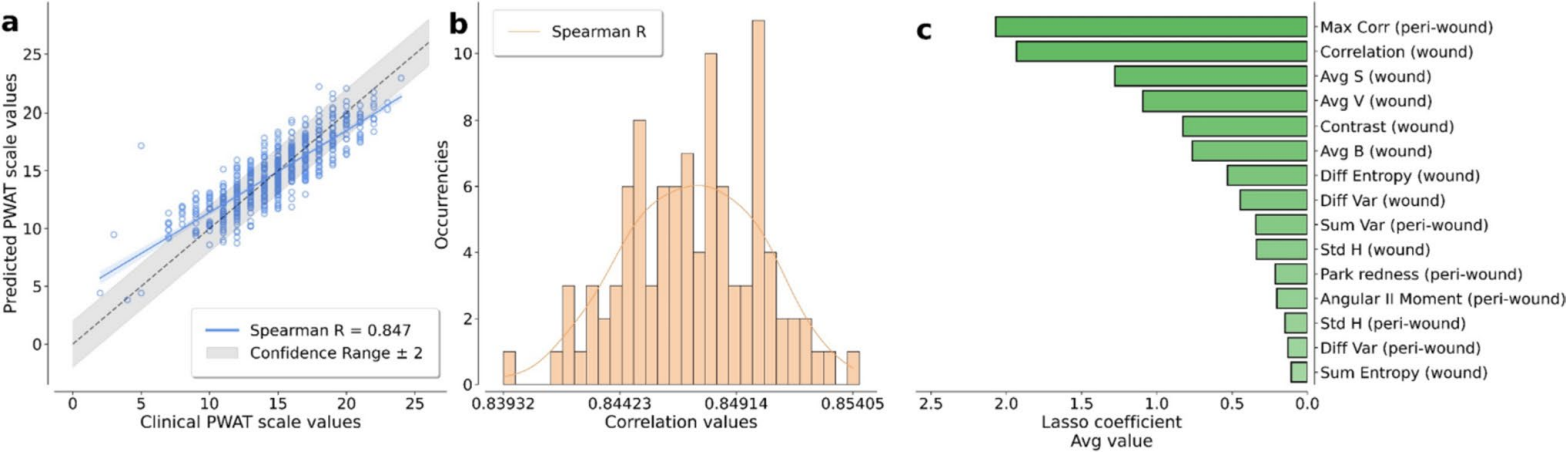
Results of the penalized regression model for predicting the PWAT scale values developed starting from the extracted features. The correlation between the ground truth and the predicted values is estimated using Spearman's rank correlation coefficient (ref. plot legends). **a** Results on a single cross-validation of the model. With the dashed line, we highlight the axes bisector corresponding to a perfect prediction. The model tends to overestimate the low PWAT scale values due to the few samples characterized by this condition. We remark that the predictions are performed on a data set independent of the training set. **b** Results obtained by the same pipeline on 100 different cross-validations. A tenfold cross-validation was applied in each iteration to estimate Spearman's rank correlation coefficients. **c** Top ranking features involved in the prediction of PWAT scores. The informative power of the features was estimated using the coefficients of the lasso regression model

**Fig. 4:**
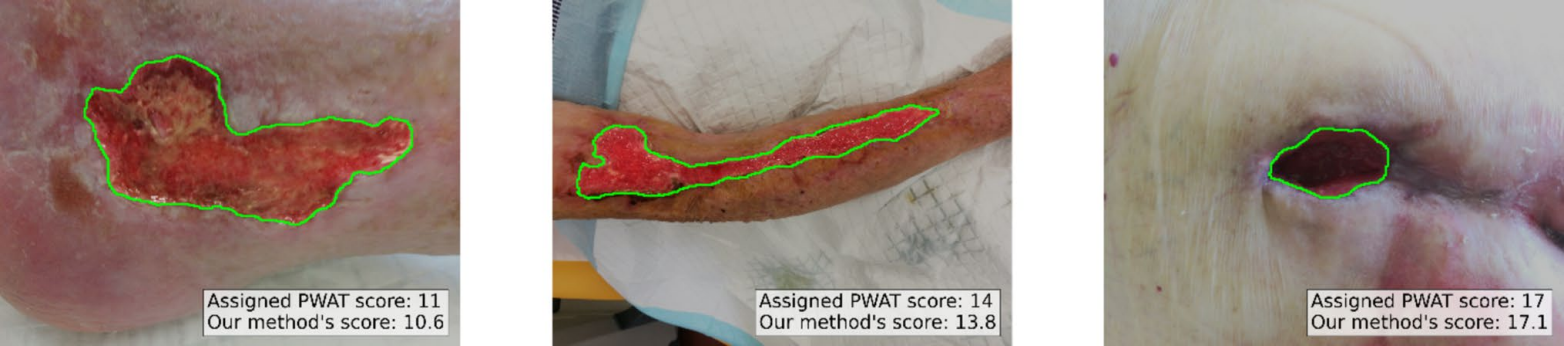
Example of the predictions obtained by the regression model on three test images. We report the assigned PWAT score and the predicted one for each image using our model. We highlighted the wound areas identified by our automated segmentation model with the green lines

We analyzed a dataset of 612 images using our automated pipeline, producing the complete set of segmentation masks, and extracting the related features. We fed a Lasso regression model using the 54 obtained features (12 color features + 2 redness features + 13 Haralick's features for both wound and peri-wound masks), estimating the correlation between the clinical PWAT values (ground truths) and the predicted ones. We trained the regression model using a tenfold cross-validation; the best model found predicts the correct PWAT scale values with a Spearman's rank correlation coefficient of 0.85 (ref. Fig. 3a) and a corresponding p-value close to zero. According to the tenfold cross validation, the correlation performances were evaluated using the test subset of the data at each fold, combining the results to obtain the score presented in the figure legend. An example of the prediction obtained on the test set is shown in Fig. 4.

We reiterated the same pipeline for 100 different cross-validations to test the robustness of our model, i.e. repeating the regression step 100 times with different train/test subdivision of the data. We re-trained a Lasso regression using tenfold cross-validation at each iteration, monitoring the model's sensitivity to different training set subdivisions. The resulting distribution of Spearman's rank correlation coefficient is shown in Fig. 3b.

We evaluated the informative power of each feature independently, performing a second set of 100 hold-out (90/10) cross-validations using the proposed pipeline, monitoring the coefficients of the Lasso regression model. The ranked distribution of the average coefficients associated with the corresponding feature is shown in Fig. 3c.

## Discussion

The automated segmentation model, combined with the refinement image processing step proposed in this work, allowed the extraction of quantitative information on both the wound and peri-wound areas. Starting from the definition proposed in the literature about the items related to the PWAT score, we extracted a series of features to characterize the wound and peri-wound areas. Each proposed feature was designed to model a different aspect of the wound area and a related PWAT sub-item. In this work, we focused on the "global" estimation of the PWAT score, but the correlation between each feature and the theoretical PWAT sub-items will be analyzed in a future work.

The results obtained on the PWAT prediction highlight a statistical agreement between (a subset of) the features extracted from the wound area and the grading scores. The robustness of the predictions on a set of images sampled with a no-rigid acquisition protocol confirms its possible use in clinical practice as a viable decision support system for dermatologists. We would like to stress that the results proposed in this work were obtained by a rigid train-test subdivision of data, i.e., evaluating the model on a never-seen set of data. Moreover, the entire pipeline produces real-time prediction on standard hardware, making it suitable for standard clinical practice and a valid candidate for smartphone implementation.

The proposed penalized regression model combines the extracted features, finding the optimal weights, i.e., parameters, to associate with each one. Beyond the resulting performances, interpreting the regression coefficients allows to rank the extracted features according to their informative power for the PWAT estimation. As expected, not all the features are equally informative, but only 15 provide information on the PWAT score. It is interesting to notice how the most informative features selected by our model involve textural measures of the peri-wound and wound areas in a fairly balanced contribution (ref. Fig. 3c), followed by the values related to the exposition and contrast of the wound. The same measures are strictly related also to the human perception of the image and its colors. This result confirms the efficiency of a Radiomic approach in medical image evaluation and the possibility to apply analogous techniques also to photographic medical images. The importance of contrast-based features could be mainly imputed to the necrotic condition of the most severe lesions which lead to a heterogeneous spread of the image colors. It is also interesting to notice how the classical redness expected in a lesion status, quantified by the Park et al. score, plays quite a negligible role in the final prediction. This behavior could be due to a bias in our dataset related to an unbalanced representation of the lesions, corresponding to different severity grades and color shades.

In our analysis, we intentionally discarded the wound area feature for the PWAT estimation; despite this information being included in the clinical practice and in the PWAT estimation, its automated computation requires a pre-determined rigid standardization of image acquisition, which could disfavor its applicability to routine clinical examinations. The *Deepskin* dataset includes wounds belonging to several anatomical positions, with images acquired without strict standardization. Therefore, the correct estimation of the wound area is impossible without a term of comparison or a pre-determined reference. We are currently developing an ad hoc segmentation model to address this issue without losing the easy-to-use characteristics of the proposed method, which will be discussed in future work.

The main limit of our work could be imputed to the monocentric source of the data and to the intrinsic bias duced by the reduced patient heterogeneity of the Italian country. A deeper validation of our system could be achieved with the analysis of a large scale multi-center dataset, involving patients with a wider heterogeneity.

A second limit of the study could be attributed to a bias in the considered PWAT scores and patients. In the analyzed dataset, the PWAT scores ranged from a minimum of 2 to a maximum of 24, lacking the scores from 25 to 32 with an unbalanced subdivision of the value classes. While this reflects real-life values ​​of the Italian population, it could nevertheless represent a limitation for the training of our system.

A further bias could be related toalso be present regarding the general picture acquisition condition. Capturing the images with a wider range of devices different light conditions could improve the robustness of the proposed method, as well as the introduction of standardized image processing techniques as preprocessing step of our analysis [31, 32].

All the limits identified in this manuscript will be faced in future works, which will manage to improve the image processing pipeline and enlarge the dataset with new records according to the clinical availability.

## Conclusions

This work introduced a fully automated pipeline for predicting the PWAT grading scale. We combined a previously published automated pipeline to analyze wound images with a feature extraction approach to quantify information related to the wound healing stage. We performed a robust machine learning analysis of the image features, providing a regression model to correctly predict the PWAT score with a Spearman's correlation coefficient of 0.85. Moreover the proposed regression model could provide PWAT predictions with a continuous range of values, i.e. floating-point scores. The possibility to describe the wound severity using a finer-grained scale could provide a better patients stratification while preserving the same informative power as the original PWAT scale.

A penalized regression model allowed us to deeply investigate the informative power of each feature extracted, providing a ranking of them according to their relation to the PWAT score. We proved that Haralick's features play a statistically significant role in the PWAT prediction. Furthermore, the features extracted on the peri-wound areas were as informative as the wound ones. This confirms the importance in defining the correct shape and boundaries of the wound area for the correct automatization of the PWAT analysis.

The proposed pipeline is currently used in the Dermatological Unit of IRCCS Sant'Orsola-Malpighi University Hospital of Bologna in Italy, and it is still being perfected to overcome the current limitations of the method. These improvements will be the subject of future work.

## Data Availability

The data used during the current study are available from the corresponding author on reasonable request. The pre-trained model for image segmentation is available in the repository, *Deepskin* (https://github.com/Nico-Curti/Deepskin). The regression model used for the PWAT estimation is available in the repository, *Deepskin* (https://github.com/Nico-Curti/Deepskin).
